# Numerical Simulation of Fluid Flow, Solidification, and Solute Distribution in Billets under Combined Mold and Final Electromagnetic Stirring

**DOI:** 10.3390/ma17020530

**Published:** 2024-01-22

**Authors:** Zhenhua Feng, Guifang Zhang, Pengchao Li, Peng Yan

**Affiliations:** 1 Faculty of Metallurgy and Energy Engineering, Kunming University of Science and Technology, Kunming 650093, China; fengzhenhua666@126.com (Z.F.); 13696392999@163.com (P.L.); 2 Linyi Iron and Steel Investment Group Special Steel Co., Ltd., Linyi 276000, China

**Keywords:** numerical simulation, billets, electromagnetic stirring, fluid flow, carbon segregation

## Abstract

In this study, a three-dimensional segmented coupled model for continuous casting billets under combined mold and final electromagnetic stirring (M-EMS, F-EMS) was developed. The model was verified by comparing carbon segregation in billets with and without EMS through plant experiments. The findings revealed that both M-EMS and F-EMS induce tangential flow in molten steel, impacting solidification and solute distribution processes within the billet. For M-EMS, with operating parameters of 250A-2Hz, the maximum tangential velocity (velocity projected onto the cross-section) was observed at the liquid phase’s edge. For F-EMS, with operating parameters of 250A-6Hz, the maximum tangential velocity occurred at $f_{l}=0.7$. Furthermore, F-EMS accelerated heat transfer in the liquid phase, reducing the central liquid fraction from 0.93 to 0.85. M-EMS intensified the washing effect of molten steel on the solidification front, resulting in the formation of negative segregation within the mold. F-EMS significantly improved the centerline segregation issue, reducing carbon segregation from 1.15 to 1.02. Experimental and simulation results, with and without EMS, were in good agreement, indicating that M+F-EMS leads to a more uniform solute distribution within the billet, with a pronounced improvement in centerline segregation.

## 1. Introduction

Electromagnetic stirring (EMS) is a widely used metallurgical process in continuous casting processes. It utilizes electromagnetic forces to stir molten steel, improving the fluidity of the steel, promoting the uniform distribution of solute elements, refining the solidification structure of the cast billet, and ultimately achieving better metallurgical results, in turn, enhancing production efficiency and product quality [1]. EMS is generally categorized into different types based on its installation location, including mold electromagnetic stirring (M-EMS), strand electromagnetic stirring (S-EMS), and final electromagnetic stirring (F-EMS). Each type of EMS has distinct metallurgical effects. Therefore, it is crucial to investigate the impact of combined EMS on the metallurgical behavior of billets to improve their quality.

Many studies based on production experiments have demonstrated that M-EMS primarily serves to refine grain structure and, to some extent, alleviate central segregation issues in strands. Wu et al. [2] investigated the influence of M-EMS on the solidification structure of strands, and their findings indicate that increasing electromagnetic torque can refine grain size, expand the equiaxed grain zone, and improve central segregation issues. Regarding the effects of F-EMS, several production experiments have shown a significant improvement in central segregation issues [3,4], and it also offers some improvement in addressing central shrinkage concerns [5]. In addition, the experimental study by Falkus et al. [6] showed that casting parameters such as casting speed also have a very obvious impact on macrosegregation.

Due to the high temperature and opacity constraints of the continuous casting process, the flow, heat transfer, and species transfer processes of molten steel cannot be directly observed through experimental methods. Therefore, numerical simulation methods serve as an ideal tool to investigate the impact of EMS on the metallurgical behavior of continuous casting strands. Since the 1960s, Flemings and his colleagues [7,8,9] have conducted pioneering research, discovering the significance of convection in the mushy zone during alloy solidification and deriving fundamental equations describing macrosegregation induced by interdendritic flow. Mehrabian et al. [10] developed a macrosegregation model considering the influence of shrinkage and thermal buoyancy on liquid flow, treating the solid–liquid two-phase region as a porous medium and calculating the flow velocity of interdendritic liquid using Darcy’s Law. The model made assumptions of numerical values for temperature gradients and solidification rates due to the absence of solving the energy transfer equation. Fuji et al. [11] attempted to solve the momentum and energy equations in the solid–liquid two-phase region but did not couple the transport phenomena between the two-phase region and the solid region, and they specified the location of the solid–liquid interface. In the early 1980s, Ridder et al. [12] reported the first macrosegregation model that explained the coupling flow between the mushy zone and the liquid region. They solved the coupled equations given by Darcy’s Law, the energy equation in the mushy zone, the Local Solute Redistribution Equation (LSRE), and the momentum and energy equations in the fully liquid region. The predicted macrosegregation patterns showed good agreement with experimental measurements. Based on these theories and models, many researchers have investigated macrosegregation behavior in various alloy systems [13,14,15]. Bennon et al. [16] studied dendrite erosion in the mushy zone and the formation of channel-type “A” segregation using a continuum model, achieving predictive capabilities by fully coupling the solute conservation equation with the energy and momentum conservation equations. Hebditch [17] examined the influence of interdendritic liquid density changes during solidification using Pb–Sn and Sn–Zn alloys and identified interdendritic convection as the primary mechanism for macrosegregation formation. The studies mentioned above mainly focused on macrosegregation in ingots, which is more complex compared to the transport behavior and formation mechanism of macrosegregation in the continuous casting system.

Many scholars have established corresponding models for different phenomena in the continuous casting process. Grundy et al. [18] proposed that hard secondary cooling significantly reduces macrosegregation through numerical simulation methods. Rajiah et al. [19] proposed that macrosegregation happens as a result of the breakage of columnar dendrites in the low ductility region of steel between zero ductile temperature (ZDT) and zero strength temperature (ZST). Melo et al. [20] calculated Secondary Dendrite Arm Spacing and second-phase particles were included, and the measured values are in good agreement with the calculated values. Mramor et al. [21] used the Reynolds-Averaged Navier–Stokes (RANS) model to predict the solute distribution within the crystallizer at different casting temperatures. The results indicated that lower casting temperatures favor a more uniform distribution of solutes. Moreover, in their study [22], a comparison was made between the Large Eddy Simulation (LES) model and the two-equation Low Re *k*−*ε* turbulence RANS model in terms of temperature, velocity, and computational times. The LES model successfully captures the transient nature of vortices, a feature that RANS-type turbulence models struggle to address. However, it is important to note that the computational cost of LES models is significantly higher compared to RANS models. Wu et al. [23] studied the solute migration process in the vicinity of the mold, and the results of this model indicate a significant influence of M-EMS on dendritic growth and solute transport during the initial solidification process of molten steel. The research suggests that the addition of M-EMS leads to a thinner solidification shell at the outlet of the mold, and due to the enhanced scouring effect of molten steel on the solidification front, it results in negative segregation. This was also proposed in the work of Kihara et al. [24].

The combined effects of M+F-EMS are also documented. Zhang et al. [25] employed a 2D–3D hybrid model to describe the influence of M+F-EMS on macrosegregation behavior in billets. In the regions of M-EMS and F-EMS, a three-dimensional model was used, while a two-dimensional model using the slicing method was applied in the secondary cooling zone. The study results reveal that the macrosegregation behavior of carbon, sulfur, manganese, and phosphorus in the mold is very similar. As the distance from the surface of the strand increases, the degree of solute segregation changes from positive to negative. Due to the significant challenge in simulating computational efficiency and convergence using geometric models of the same size as the continuous casting machine, there are fewer reports on using full three-dimensional models to describe the metallurgical effects of combined EMS. Wang et al. [26] established a curved three-dimensional model for the macrosegregation of billets with M+F-EMS. The model neglects the effect of thermal solute buoyancy, and it has been reported that the calculation time for this model is approximately 50 days. Dong et al. [27] developed a three-dimensional model that ignores the curvature of the continuous casting machine. Although the difference in geometric models may lead to inaccurate results, the study also demonstrated the positive impact of M-EMS+F-EMS on improving the solute distribution in strands.

This study, following the geometry of a continuous casting machine in use at a steel plant, constructed a segmented three-dimensional multiphysics coupling model for the curved continuous casting of billets. The primary objective was to investigate the influence of M+F-EMS on the internal fluid dynamics, heat transfer, solidification, and solute distribution in billet. Subsequently, the production experiments were conducted at the steel plant under two EMSs and non-EMS conditions. The results of these experiments were used to validate the model, particularly with regard to carbon segregation in the experimental billets.

## 2. Model Description

### 2.1. Assumptions

(1) Molten steel is considered to be an incompressible Newtonian fluid, and all thermophysical properties are assumed to be uniform and isotropic [27].
(2) The continuous casting process is assumed to be in a steady-state or quasi-steady-state condition. This means that within the computational domain, physical parameters such as flow state, temperature distribution, and solute distribution do not vary with time or vary periodically.
(3) The effects of mold taper and vibration, as well as phenomena like solidification shrinkage and bulging, are neglected. It is assumed that the shape of the cast billet remains constant throughout the entire continuous casting process.
(4) Low Reynolds number turbulence models are employed to simulate the flow field, in accordance with previous studies [27,28].
(5) This study does not account for the electromagnetic heat generated by EMS on the cast billet.
(6) Due to the similar segregation behaviors of solute elements such as phosphorus, sulfur, and manganese in steel, this study specifically focuses on the macrosegregation behavior of carbon. Additionally, interactions between different elements are disregarded.

### 2.2. Governing Equations

#### 2.2.1. Fluid Flow

The fluid flow can be described by the following governing equations:

(1) $$\nabla \cdot \left(\rho u\right)=0$$

where ρ represents the density of the mixture, kg/m^3^, and u represents the velocity of the mixture, m/s.

(2) $$\nabla \cdot \left(\rho uu\right)=\nabla \cdot \left(\mu_{eff}\nabla u\right)-\nabla p+\rho g+F_{mag}+F_{b}+S_{D}$$

where p represents the pressure, Pa. g stands for the acceleration due to gravity, which, in this study, is set to 9.81 m/s^2^. $F_{mag}$ denotes the electromagnetic force, and its description is provided in the electromagnetic governing equations. $\mu_{eff}$ represents the effective viscosity, which is calculated as the sum of the laminar viscosity coefficient μ and the turbulent viscosity coefficient $\mu_{T}$. The value of the turbulent viscosity coefficient $\mu_{T}$ can be determined using the following equation:

(3) $$\mu_{T}=\rho C_{\mu }\frac{k}{\varepsilon^{2}}$$

where $C_{\mu }$ is an empirical constant with a value of 0.09. k represents the turbulent kinetic energy, m^2^/s^2^. ε stands for the turbulent dissipation rate, m^2^/s^3^.

In Equation (2), $F_{b}$ represents the thermal and solutal buoyancy and can be calculated using the following formula:

(4) $$F_{b}=\rho g[\beta_{T}\left(T-T_{ref}\right)+\beta_{C}\left(C-C_{ref}\right)]$$

where $\beta_{T}$ denotes the thermal expansion coefficient, 1/K. T represents the temperature, K. $T_{ref}$ is the reference temperature, assumed as the liquidus temperature in this study. $\beta_{c}$ stands for the solutal expansion coefficient, 1/wt.%. C signifies the carbon concentration, 1/wt.%. $C_{ref}$ is the reference carbon concentration, representing the initial carbon content in the molten steel.

In this study, the enthalpy-porosity technique is employed to treat the mushy zone as a porous medium. In Equation (2), $S_{D}$ represents the Darcy source term and can be computed using the following formula:

(5) $$S_{D}=\frac{(1-\alpha {)}^{2}}{\alpha^{3}+\xi }A_{mush}\left(u-u_{p}\right)$$

where α denotes the liquid phase fraction. ξ is a very small positive number, chosen to ensure that the denominator is not zero (in this study, it takes a value of 0.001). $u_{p}$ represents the casting speed, m/s. $A_{mush}$ stands for the mushy zone constant, and its value can be calculated using the following formula [29]:

(6) $$A_{mush}=\frac{180}{\lambda_{2}^{2}}$$

(7) $$\lambda_{2}=\left\{\begin{array}{c} \;\left(169.1-720.9\cdot C_{C}\right)\cdot C_{R}\;0<C_{C}<0.15\\ 143.9\cdot C_{R}^{-0.3616}\cdot C_{C}^{\left(0.5501-1.996C_{C}\right)}\;0.15<C_{C}\; \end{array}\right.$$

where $\lambda_{2}$ represents the secondary dendrite arm spacing, μm. $C_{R}$ stands for the cooling rate, °C/s; in this work, the cooling rate was calculated by taking the difference between the cross-sectional average temperature at the solidification endpoint and the pouring temperature, divided by the time taken to reach the solidification endpoint and the value is 0.7 °C/s in this work. $C_{C}$ represents the carbon content, wt.%.

In this study, the low Reynolds number turbulent k–ε model is employed, where the turbulent kinetic energy k and turbulent dissipation rate ε in Equation (3) are determined using the following expressions:

(8) $$\rho \left(u\cdot \nabla \right)k=\nabla \cdot \left[\left(\mu +\frac{\mu_{T}}{\sigma_{k}}\right)\nabla k\right]+P_{k}-\rho \varepsilon$$

(9) $$\rho \left(u\cdot \nabla \right)\varepsilon =\nabla \cdot \left[\left(\mu +\frac{\mu_{T}}{\sigma_{\varepsilon }}\right)\nabla \varepsilon \right]+C_{\varepsilon 1}\frac{\varepsilon }{k}P_{k}-C_{\varepsilon 2}\rho \frac{\varepsilon^{2}}{k}$$

where $P_{k}$ represents the turbulent kinetic energy generated due to the mean velocity gradient, m^2^/s^2^. $\sigma_{k}$ is the Prandtl number for turbulent kinetic energy k, with a value of 1.0 in this study. $\sigma_{\varepsilon }$ is the Prandtl number for turbulent dissipation rate ε, set to 1.3 in this study. $C_{\varepsilon 1}$ and $C_{\varepsilon 2}$ are empirical constants in the low Reynolds number turbulent k–ε model, taking values of 1.44 and 1.92, respectively, in this study.

#### 2.2.2. Electromagnetism

In this study, the frequency domain method is used to calculate the electromagnetic fields generated by M and F-EMS. In the frequency domain, the relationship between electric field and magnetic induction intensity is converted by Fourier transform into the following equations:

Faraday’s Law of Electromagnetic Induction:

(10) ∇×E=−jωB

where E represents the electric field strength, N/C. j represents the imaginary unit. ω represents the angular frequency, rad/s. B represents the magnetic flux density, T.

Gauss’s Law for Magnetic Fields:

(11) ∇·B=0

Ampere’s Law with Maxwell’s Addition:

(12) $$\nabla \times H=J+j\omega \varepsilon_{r}E$$

where H represents the magnetic field strength, A/m. J represents the current density, A/m^2^.

Without considering the influence of molten steel flow on the magnetic field, Ohm’s Law can be simplified to the following form:

(13) J=σE

where σ represents the electrical conductivity, S/m.

The constitutive equation for the above formulae is:

(14) B=μH

where μ represents the magnetic permeability, H/m.

The relative permeability of iron core is set to 1000, the relative permeabilities of air, strand, copper mold, and coil are set to 1, the electric conductivity of strand is set to 7.14 × 10^5^ S/m, and the electric conductivity of copper mold is set to 3.18 × 10^7^ S/m.

The time-averaged Lorentz force generated by EMS can be calculated using the following formula:

(15) $$F_{mag}=\frac{1}{2}Re\left(J\times B^{*}\right)$$

where Re represents the real part of a complex number and $B^{*}$ is the complex conjugate of the magnetic induction vector B.

#### 2.2.3. Heat Transfer and Solidification

In the continuous casting system, the energy conservation equation during the solidification process of billets is expressed as follows:

(16) $$\nabla \cdot \left(\rho uH\right)=\nabla \cdot \left(k_{eff}\nabla T\right)$$

where H represents the total enthalpy, J/kg. $k_{eff}$ denotes the effective thermal conductivity, W/(m·K). H and $k_{eff}$ can be expressed by the following formulae, respectively:

(17) $$H=h_{ref}+\int_{T_{ref}}^{T}c_{p}dT+f_{l}L$$

(18) $$k_{eff}=\left\{\begin{array}{l} k_{T,s}\;T\leq T_{s}\\ k_{T,s}f_{s}+k_{T,l}f_{l}\;T_{s}<T<T_{l}\\ k_{T,l}+\frac{\mu_{T}}{{Pr}_{t}}\;T>T_{l} \end{array}\right.$$

where $h_{ref}$ represents the enthalpy at the reference temperature, J/kg. $c_{p}$ is the specific heat capacity of steel, J/(kg·K). L is the latent heat of steel, J/kg. ${Pr}_{t}$ is the turbulent Prandtl number. $f_{l}$ and $f_{s}$ are the liquid and solid phase fractions, and they can be calculated using the following formulae [30]:

(19) $$f_{l}=1-f_{s}=\left\{\begin{array}{c} \;0\;T\leq T_{s}\;\\ \frac{T-T_{s}}{T_{l}-T_{s}}\;T_{s}<T<T_{l}\\ 1\;T_{l}\leq T \end{array}\right.$$

where $T_{l}$ is the liquidus temperature, set as a constant in this study at 1788 K and $T_{s}$ is the solidus temperature, also set as a constant in this study at 1738 K.

#### 2.2.4. Solute Transport

In this study, the following equations are used to describe the carbon transport process within the billet:

(20) $$\nabla \cdot \left(\rho uC\right)=\nabla \cdot \left[\rho \left(D_{l}+\frac{\mu_{T}}{{Sc}_{t}}\right)\nabla \cdot C\right]+\nabla \cdot \left[\rho f_{s}D_{s}\nabla \cdot \left(C_{s}-C\right)\right]+\nabla \;\;\cdot \left[\rho f_{l}D_{l}\nabla \cdot \left(C_{l}-C\right)\right]-\nabla \cdot \left[\rho \left(u-u_{cast}\right)\left(C_{l}-C\right)\right]$$

where C is the carbon concentration, wt.%. ${Sc}_{t}$ is the turbulent Schmidt numbers, set to 1. $D_{s}$ is the diffusion coefficient of carbon in the solid phase, m^2^/s. $C_{s}$ is the carbon concentration in the solid phase, wt.%. $D_{l}$ is the diffusion coefficient of carbon in the liquid phase, m^2^/s. $C_{l}$ is the carbon concentration in the liquid phase, wt.%. $C_{s}$ and $C_{l}$ can be expressed by the following equations:

(21) $$C_{l}=\frac{C}{1+f_{s}\left(k_{C}-1\right)}$$

(22) $$C_{s}=\frac{k_{c}C}{1+f_{s}\left(k_{C}-1\right)}$$

where $k_{c}$ is the equilibrium distribution coefficient for carbon.

## 3. Computational Procedure

### 3.1. Geometry and Meshing

This study employed COMSOL Multiphysics 5.6 (COMSOL, Inc., Burlington, VT, USA) and SOLIDWORKS 2018 (Dassault Systèmes SOLIDWORKS Corp., Waltham, MA, USA) to establish a three-dimensional geometric model of a bent continuous casting billet. The geometric model of the continuous casting billet was created using COMSOL Multiphysics. The three-dimensional geometric models of M-EMS and F-EMS were constructed using SOLIDWORKS. These models were exported in the .igs file format and imported into COMSOL. The positions of M-EMS and F-EMS in COMSOL were adjusted based on the installation locations in the steel plant. The F-EMS was aligned parallel to the cross-section of the cast billet at its installation location. The curved three-dimensional model was established according to the dimensions of a 10-strand continuous casting machine used in a specific steel plant. The model utilizes a straight-type SEN at the mold inlet, consistent with the practical production setup. The SEN has an inner diameter of 0.026 m, an outer diameter of 0.09 m, and is submerged to a depth of 0.11 m. The installation positions of the stirrers and other geometric parameters are detailed in Table 1.

**Figure 1:**
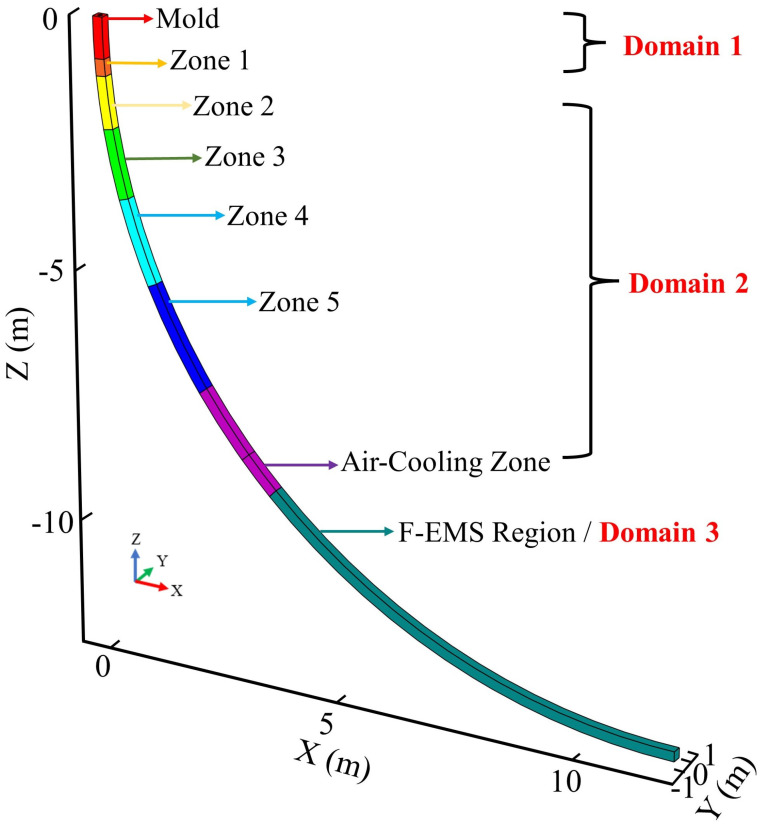
Schematic of computational domain division.

This study divides the three-dimensional multiphysics coupled model into three computational domains based on the flow and solute transport phenomena in different regions during the continuous casting process. The geometric model division method is illustrated in Figure 1. The mold region and Zone 1 of the secondary cooling zone are grouped as Domain 1. This is due to the consideration of the effect of M-EMS and the jet action of molten steel entering the mold at a high speed from the submerged entry nozzle (SEN), leading to the formation of forced convection in this area. Domain 2 is defined by dividing Zone 2 to Zone 5 of the secondary cooling zone and a portion of the air-cooling zone. This division is motivated by the fact that after the molten steel exits the forced convection zone, its flow is mainly driven by gravity in this region, where convection is primarily induced by thermal and solutal buoyancy. Including a portion of the air-cooling zone in Domain 2 is to optimize computational resources and enhance efficiency since significant computational resources are required for magnetic field and electromagnetic force calculations. The outlet of Domain 2 is set approximately 1 m away from the length of F-EMS to conserve computational resources. Domain 3 is designated for the F-EMS action region, focusing on the forced convection effect induced by F-EMS.

The segmented model is computed by sequentially solving three domains, where the physical quantities (including components of velocity in X, Y, Z denoted as u, v, w, as well as turbulent model variables k and ε, temperature T, and solute concentration C) at the outlet of the preceding domain serve as the boundary conditions for the inlet of the subsequent domain. The steady-state method is employed for fluid flow, heat transfer, solidification, and solute transport behaviors of the billet without EMS. When EMS is introduced, a frequency domain method is initially used to calculate the electromagnetic field and electromagnetic forces. Subsequently, a frequency domain steady-state method is employed to calculate the fluid flow, heat transfer, solidification, and solute transport behaviors influenced by EMS. All simulation computations are conducted using COMSOL Multiphysics.

Due to the curvature of the model, the direction and magnitude of the casting speed vary with position. As is evident from Figure 1, the cast billet is symmetric about the *Y*-axis. Therefore, the casting speed does not have a component along the *Y*-axis ($u_{cast-Y}=0$). The components of the casting speed along the *X*-axis and *Z*-axis are calculated by the following equations:

(23) $$u_{cast-X}=u_{cast}\cdot sin\theta$$

(24) $$u_{cast-Z}=u_{cast}\cdot cos\theta$$

(25) $$\theta =\arcsin\left(\frac{s}{R}\right)$$

where $u_{cast-X}$ is the component of the casting speed along the *X*-axis, m/s. $u_{cast-Z}$ is the component of the casting speed along the *Z*-axis, m/s. θ is the angle between the position and the meniscus. s is the vertical distance from this position to the meniscus, equal to the absolute value of the *Z*-axis coordinate of the position, m. R is the straight-line distance from the position to the center of the curved continuous casting machine, m.

**Figure 2:**
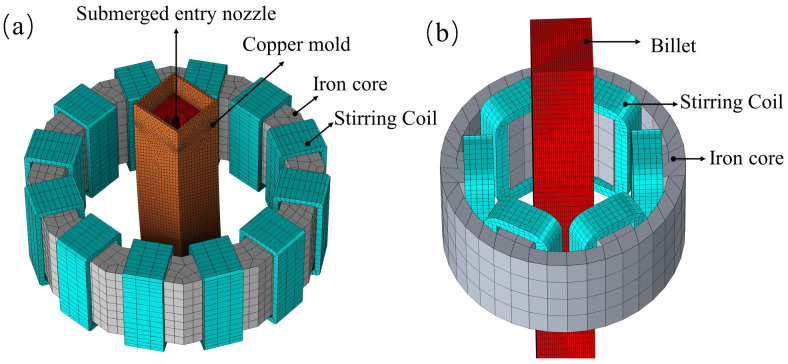
Mesh division of (**a**) M-EMS region; (**b**) F-EMS region.

Figure 2 displays the geometric model and meshing of the regions affected by M-EMS and F-EMS. M-EMS comprises 12 coils, divided into six groups, with each group carrying current in the same phase. The phase difference between each group is 120°. F-EMS consists of 6 coils and, thus, no grouping is needed. The three-phase current input method is the same as that of M-EMS. Hexahedral meshing is employed for both the billet and the electromagnetic stirrer. However, when calculating the magnetic field generated by EMS, meshing is applied to the surrounding air domain. Due to the complexity of the geometric model, adaptive tetrahedral meshing is used for meshing the air domain and the copper mold. The total number of meshes for the three computational domains is approximately 3.5 million, and the installation positions of M-EMS and F-EMS are listed in Table 1.

### 3.2. Boundary Conditions

#### 3.2.1. Inlets and Outlets

According to the calculation method of the segmented model, the entrance boundary conditions for Domain 2 and Domain 3 are the physical quantities at the outlet of the previous computational domain. Therefore, it is only necessary to provide the boundary conditions at the entrance of Domain 1. The entrance of Domain 1 is the upper end of the SEN, and the values of the physical quantities required for the turbulence model at this location can be calculated using the following formulae:

(26) $$u_{0}=\frac{4S^{2}}{\pi d^{2}}u_{cast}$$

(27) $$k_{0}=0.01\cdot {u_{0}}^{2}$$

(28) $$\varepsilon_{0}=\frac{{k_{0}}^{1.5}}{d}$$

where $S^{2}$ is the cross-sectional area of the cast billet, m. d is the diameter of the SEN, m. $u_{cast}$ is the casting speed, m/s. The molten steel temperature at the inlet is set to the pouring temperature, with a value of 1813 K. The carbon concentration of the steel at the inlet is set to 0.20 wt.%. The casting speed in all three computational domains is set to 1.4 m/min.

The outlets of all three computational domains are uniformly set to a fully developed flow, meaning that the normal gradients of all variables are set to zero.

#### 3.2.2. Walls

The surface of the strand is designated as a slip boundary condition in the fluid flow calculations. In the heat transfer computations, the surface is subjected to heat flux coefficient conditions. Specifically, in the heat transfer calculations within the mold segment, the wall heat flux $q_{m}$ is determined using the following formula:

(29) $$q_{m}=\rho_{w}c_{w}W_{m}\frac{{∆T}_{w}}{A_{m}}$$

where $\rho_{w}$ represents the density of the cooling water, kg/m^3^. $c_{w}$ denotes the specific heat capacity of the cooling water, J/(kg·K). $W_{m}$ stands for the flow rate of the cooling water in the mold, L/min. ${∆T}_{w}$ signifies the temperature difference between the inlet and outlet of the cooling water, K. $A_{m}$ represents the contact area between the billet and the mold, m^2^.

The heat flux in the secondary cooling zone is set as $q_{s}$ and is determined by the following formula:

(30) $$q_{s}=h_{s}(T_{surf}-T_{w})$$

where $T_{surf}$ represents the surface temperature of the cast billet, K. $T_{w}$ is the temperature of the cooling water, K. $h_{s}$ is the heat transfer coefficient. The magnitude of $h_{s}$ is correlated with the cooling water flow rate for each segment of the secondary cooling zone and is calculated using the following formula [31]:

(31) $$h_{s}=116+10.44{W_{s}}^{0.851}$$

where $W_{s}$ denotes the cooling water flow rate in the secondary cooling zone, L/min. The lengths and cooling water flow rates for each segment of the secondary cooling zone are listed in Table 2.

The heat flux in the air-cooling zone is set as $q_{a}$, and its magnitude is determined by the following formula:

(32) $$q_{a}=\varepsilon_{s}\sigma \left(T_{surf}^{4}-T_{amb}^{4}\right)$$

where $T_{amb}$ represents the ambient temperature, K. $\varepsilon_{s}$ is the emissivity of the strand and σ is the Stefan–Boltzmann constant.

#### 3.2.3. Thermal Properties

The subject of this study is billets with a cross-sectional dimension of 200 mm × 200 mm produced by a 10-strand continuous casting machine in a steel plant. The steel grade is 20# and its chemical composition is listed in Table 3. The thermal properties’ parameters used in the simulation calculations are presented in Table 4.

## 4. Results and Discussions

### 4.1. Model Validation

To validate the coupled model established in this study, carbon segregation experiments were conducted on the 10-strand continuous casting machine equipped with M-EMS and F-EMS in a steel plant. The production parameters of the steel plant are listed in Table 5.

**Figure 3:**
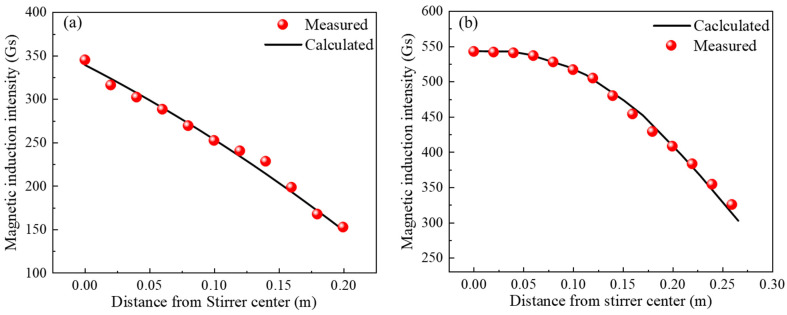
Comparison of measured and calculated magnetic induction intensity: (**a**) M-EMS; (**b**) F-EMS.

This study conducted a comparison between the measured and numerically calculated magnetic field magnitudes for M and F-EMS, as illustrated in Figure 3. Figure 3a and b, respectively, depict the comparison between the measured values and numerical simulation results of magnetic induction magnitudes at different distances from the center along the central axis of M and F-EMS. The operating parameters for M-EMS were set at 200A-3Hz, and for F-EMS at 250A-8Hz. The Tesla meter model HT201 was used for measuring magnetic induction intensity. The results indicate a good agreement between the calculated and measured values, validating the reliability of the magnetic field model employed in this study. It is important to note that both numerical simulations and experimental measurements were conducted under the condition of no billet passing through.

**Figure 4:**
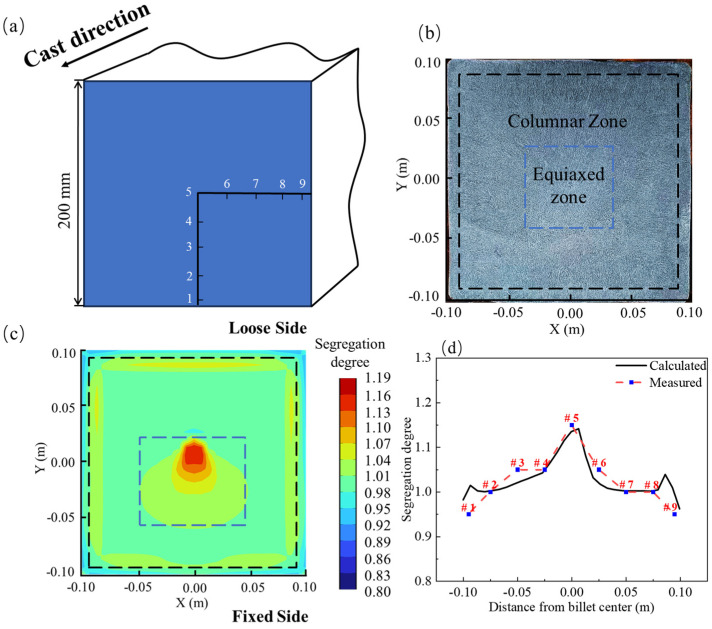
Model validation: (**a**) sampling method; (**b**) macrostructure image; (**c**) simulated carbon distribution; (**d**) comparison of simulated and experimental results.

This study conducted a simulation calculation of carbon segregation in the billet without employing EMS and compared the results with experimental data. Figure 4 illustrates the experimental sampling method, macrostructure photographs of the trial billet, simulation outcomes, and the comparison between experimental and simulation results. In the carbon segregation experiment, shavings were collected through drilling after the complete solidification of the billet. The sampling took place just after the straightening section of the continuous casting machine (after Z = −12 m). Subsequent carbon analysis was performed using a carbon–sulfur analyzer (EMIA Pro, Horiba Inc., Osaka, Japan). A 4 mm-diameter drill bit was employed to create holes on the cross-section of the billet at nine points (1 to 4, 6 to 9, with distances from the billet edge at 5 mm, 25 mm, 50 mm, and 75 mm, where 5 denotes the center), as illustrated in Figure 4a. The segregation degree “r” was used in this study to represent the extent of carbon segregation, with its value determined by the following formula:

(33) $$r=\frac{c}{c_{0}}$$

where c represents the carbon concentration at the point, wt.%. $c_{0}$ stands for the average carbon concentration at each sampling point in the experiment and is the initial carbon concentration in the molten steel in the simulation, wt.%. When r>1, it indicates positive segregation, and when r<1, it indicates negative segregation.

In the absence of EMS, Figure 4b presents the macrostructure image of the experimental billet sample after being immersed in a 1:1 hydrochloric acid–water solution at 60 °C for 10 min. Examination of the billet’s macrostructure reveals a typical solidification pattern, featuring an outermost chilled zone, an inner coarser columnar zone, and a central equiaxed zone [32]. Notably, a subtle point segregation is discernible at the billet’s central position. The carbon segregation distribution after complete solidification, as simulated, is illustrated in Figure 4c. The figure demonstrates the development of negative segregation in the corners and edges of the billet, consistent with the chilled zone depicted in Figure 4b. Additionally, a subtle positive segregation is observed in the subsurface. Significantly, there is a prominent occurrence of positive segregation in the central part, corresponding to the central equiaxed zone shown in Figure 4b. Figure 4d presents a comparison between simulation and experimental results, with the horizontal axis denoting the distance from the center of the billet and the vertical axis representing the segregation degree. The simulated sampling line aligns with the experimental one. At the center of the billet, the experimental measurement of the carbon segregation degree is 1.15, while the simulated segregation degree is 1.13, resulting in an error of less than 2%. Furthermore, the results at other measurement points exhibit good concordance with the simulation. Therefore, it can be concluded that the three-dimensional coupled model established in this study is accurate.

### 4.2. Fluid Flow and Solidification

**Figure 5:**
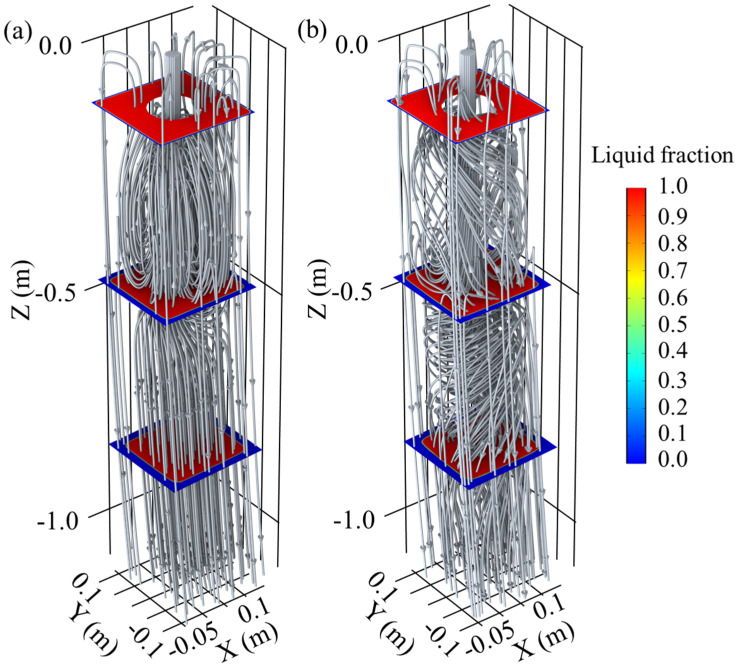
Effect of M-EMS on fluid flow and solidification: 3D stream-line distribution (**a**) without M-EMS; (**b**) M-EMS at 250A-2Hz.

The simulation results depicting the flow field and liquid fraction distribution in the M-EMS operating region are presented in Figure 5. In the absence of M-EMS, when molten steel enters the mold from the SEN, a distinct circulation movement is formed below due to the jet effect. A portion of the molten steel moves upward along the initial solidifying shell and flows back along the casting direction upon reaching the meniscus. This creates a smaller circulation around the meniscus, solidifying shell, and outer wall of the SEN. This region exhibits poorer fluidity compared to the area below the SEN, constituting a typical “dead zone” beneath the meniscus [33]. Most of the molten steel flows along the casting direction at the bottom of the circulation formed by the impact of the jet. The curved model leads to an asymmetric flow, with the depth of impact for molten steel on the fixed side being approximately 400 mm, while on the loose side, it is approximately 500 mm, as shown in Figure 5a. Simultaneously, the difference in the degree of scouring of the solidification front on the fixed and loose sides results in uneven solute distribution. Figure 5b displays the three-dimensional streamline distribution with M-EMS operating parameters at 250A-2Hz. Under the stirring effect, molten steel forms a noticeable rotational flow in the mold, primarily in the region below the SEN to the mold exit. The flow in the dead zone undergoes little change, preventing molten steel fluctuations at the meniscus that could lead to slag entrapment. Therefore, the installation position of M-EMS can be considered reasonable. The liquid fraction distributions at Z = −0.11 m (SEN outlet), Z = −0.45 m (M-EMS center), and Z = −0.80 m (mold exit) are also shown in both Figure 5a,b. Under the strong cooling conditions in the mold, a thin solidifying shell has already formed at the SEN outlet. As the position descends, the thickness of the solidifying shell gradually increases. It is noteworthy that with the addition of M-EMS, the shape of the liquid phase pool also shifts in alignment with the direction of the rotating flow.

**Figure 6:**
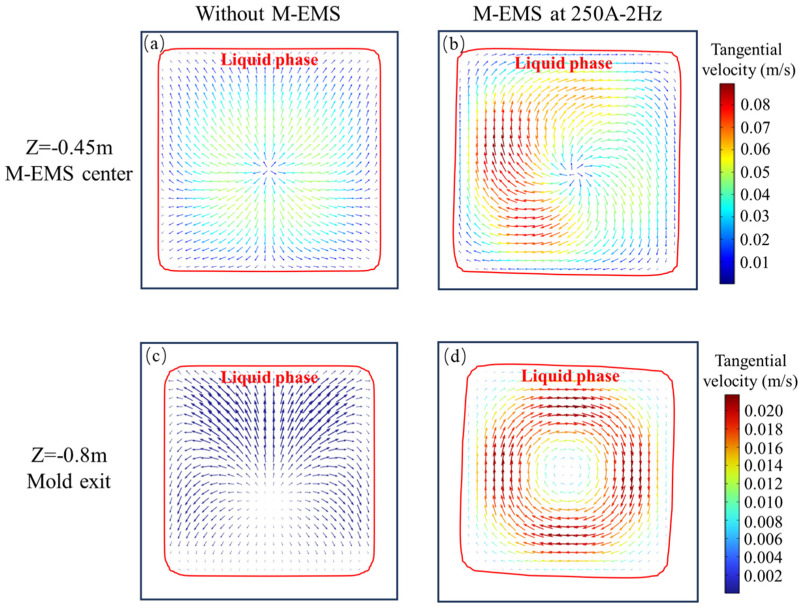
Vector plots on cross-sections: Z = −0.45 m (**a**) without M-EMS, (**b**) M-EMS at 250A-2Hz; Z = −0.8 m (**c**) without M-EMS, (**d**) M-EMS at 250A-2Hz.

In the presence and absence of M-EMS, the tangential velocity (velocity projected onto the cross-section) and the shape of the liquid phase at the M-EMS center cross-section (Z = −0.45 m) and the mold outlet (Z = −0.8 m) are shown in Figure 6. In this study, the region where $f_{l}>0.7$ is considered as the liquid phase, $f_{l}<0.3$ as the solid phase, and other regions as the mushy zone. The red line in the figure represents the contour line of $f_{l}>0.7$, indicating the shape of the liquid phase. At Z = −0.45 m, without M-EMS, the tangential velocity is mainly generated by the circulating flow formed by the jet effect, as shown in Figure 6a. When M-EMS operates at 250A-2Hz, the stirring effect of M-EMS and the jet effect of the SEN are both strong. The distribution of tangential velocity is irregular despite the trend of rotational flow, as shown in Figure 6b. The distribution of tangential velocity at the Z = −0.80 m cross-section is illustrated in Figure 6c,d. It is observed that the shape of the liquid phase has undergone a significant shift, rotating clockwise with the direction of the rotational flow. The magnitude of the tangential velocity shows a clear pattern on this cross-section, being the largest at the edges of the solidification front and smaller at the corners and the center. This phenomenon is attributed to the higher solidification resistance at the corners, lower electromagnetic force at the center, and a continued strong tendency of steel liquid flow along the casting direction.

**Figure 7:**
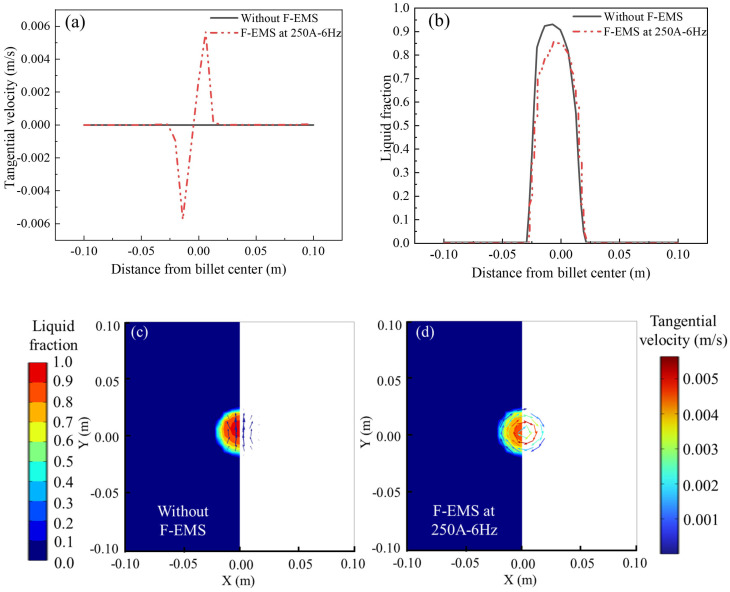
The influence of F-EMS on velocity and liquid fraction distribution: (**a**) tangential velocity; (**b**) liquid fraction; (**c**,**d**) contour plots of liquid fraction with and without F-EMS, along with vector plots of tangential velocity.

Figure 7 illustrates the impact of F-EMS on the liquid fraction and tangential velocity, with results obtained under the operation of M-EMS at 250A-2Hz. In Figure 7a, the tangential velocity on the central cross-section with and without F-EMS is compared. It can be observed that without F-EMS, there is almost no rotational convection in the molten steel, resulting in a nearly zero tangential velocity. However, with F-EMS operating at 250A-6Hz, a significant rotational flow is generated in the molten steel in the solidification end, with a maximum tangential velocity of approximately 0.006 m/s. Figure 7b compares the influence of F-EMS on the distribution of liquid fraction. It is evident that the addition of F-EMS significantly reduces the central liquid fraction, decreasing from 0.93 without F-EMS to 0.85. This reduction is attributed to F-EMS promoting convection in the late stage of solidification, thereby accelerating heat dissipation. Figure 7c,d contrast the distribution of liquid fraction and the vector plot of tangential velocity on the Z = −9.23 m cross-section with and without F-EMS. Without F-EMS stirring, the convection in the molten steel in the late stage of solidification is mainly due to the action of thermal solutal buoyancy. In the vector plot on the right side in Figure 7c, weak circulation can be observed, where the magnitude of the tangential velocity is approximately 10^−7^ m/s. This is because the size of the molten steel in the late-stage liquid phase limits the development of flow, and the decreased steel temperature leads to a smaller thermal solutal buoyancy, resulting in weak convection at this location. Figure 7d shows the situation under F-EMS with operating parameters at 250A-6Hz. A clockwise rotational flow pattern is clearly visible on this cross-section, and the maximum tangential velocity occurs at the position of the solidification front (approximately $f_{l}=0.7$). This phenomenon is attributed to the fact that both electromagnetic force and solidification resistance increase with the distance from the center of the billet. At $f_{l}=0$, the solidification resistance reaches its maximum. Under the combined action of these two forces, this phenomenon occurs.

### 4.3. Solute Distribution

**Figure 8:**
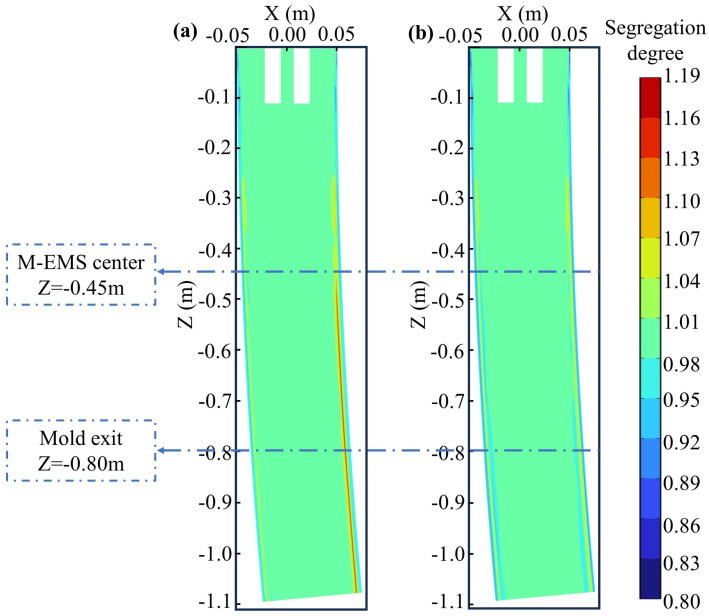
Carbon distribution on the longitudinal section of Domain 1: (**a**) without M-EMS; (**b**) M-EMS at 250A-2Hz.

In the presence and absence of M-EMS, the carbon distributions on the central longitudinal section (Y = 0) of Domain 1 are shown in Figure 8. Without EMS, due to the lower solubility of carbon in the solid phase compared to the liquid phase, carbon is expelled into the molten steel. As a result, the initial solidifying shell has a lower carbon concentration, leading to a slight negative segregation, with a segregation index of approximately 0.92. In the subsurface, the combined effect of rising circulation and thermal solutal buoyancy causes higher carbon concentration steel to gather near Z = −0.3 m. As solidification progresses and the diffusion coefficient of carbon in the solid phase is small, a positive segregation layer is formed. The solute distribution in the curved model is asymmetrical, with stronger “washing effects” on the outer arc side compared to the inner arc side. Along the outer arc side, the positive segregation is reduced due to the washing effect of molten steel, resulting in a smaller positive segregation degree compared to the inner arc side, as shown in Figure 8a. When M-EMS operates at 250A-2Hz, the solute distribution undergoes changes. The positive segregation on the inner arc side is noticeably reduced. Due to the circulation formed by M-EMS, there is a slight negative segregation layer outside the positive segregation zone below the M-EMS installation position. The negative segregation index is approximately 0.95, as shown in Figure 8b. The positions of the M-EMS center and the mold exit are marked in Figure 8. The carbon distribution results on the cross-section of Domain 1 will be compared at these two positions.

**Figure 9:**
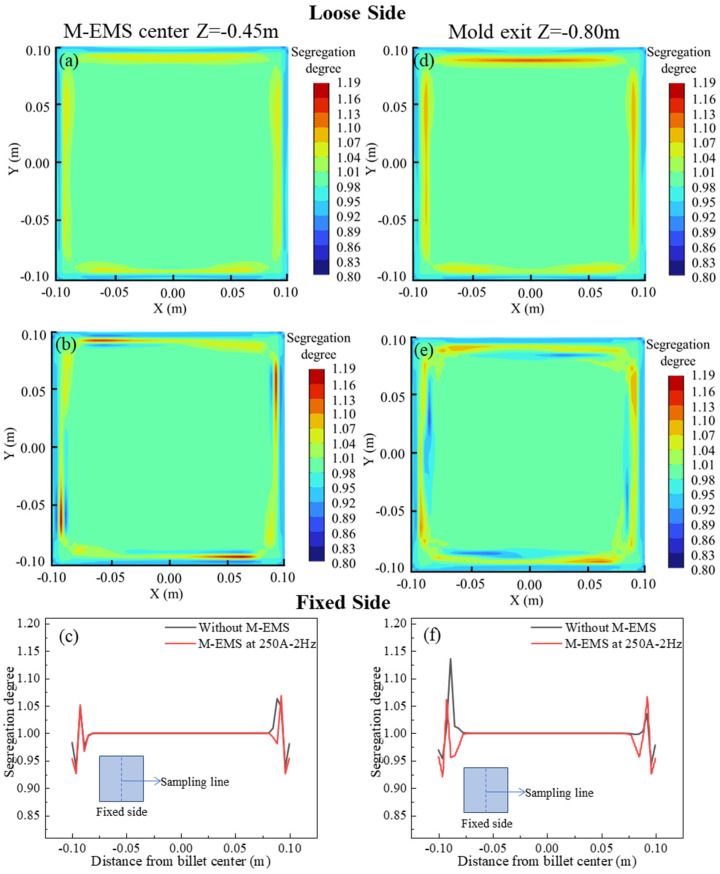
Carbon distribution on the cross-section: M-EMS center section (Z = −0.45 m) (**a**) without M-EMS; (**b**) with M-EMS; (**c**) carbon distribution along the centerline, mold exit (Z = −0.80 m); (**d**) without M-EMS; (**e**) with M-EMS; (**f**) carbon distribution along the centerline.

The carbon distributions on the central section of M-EMS and the mold outlet section are depicted in Figure 9. The carbon distributions on the cross-section at Z = −0.45 m with and without M-EMS are illustrated in Figure 9a and Figure 9b, respectively. In the absence of M-EMS, it is evident on this section that the degree of positive segregation is smaller on the fixed side compared to the loose side, as shown in Figure 9a, corresponding to Figure 8a. When M-EMS operates at 250A-2Hz, the flow pattern of the melt in the mold undergoes changes. The upward and lateral swirling flow leads to an increased degree of both positive and negative segregation, with severe segregation occurring near the corner, as depicted in Figure 9b. The comparison of the carbon segregation degree along the centerline of this cross-section is presented in Figure 9c, where negative values of X represent the side closer to the fixed side, and positive values are closer to the loose side. It can be observed from this figure that with the addition of M-EMS, the width of the positive segregation area on the loose side decreases, but a slight negative segregation appears near the solidification front, with a segregation degree of approximately 0.98. The carbon distribution on the cross-section at the mold outlet is depicted in Figure 9d,e. Without M-EMS, the carbon distribution pattern at the mold outlet is similar to that at Z = −0.45 m, but the degree of positive segregation is more severe, as shown in Figure 9d. After adding M-EMS, the positive and negative segregation degrees on this section are mitigated compared to Z = −0.45 m, as illustrated in Figure 9e. The comparison of the carbon segregation degree along the centerline of this cross-section is shown in Figure 9f. It is observed that the addition of M-EMS reduces the positive segregation, and the position of negative segregation corresponds to the flow direction, shifting clockwise. However, due to the effect of the swirling flow on the solidification front, a negative segregation layer forms in the mold, and it cannot be eliminated in the subsequent continuous casting process.

**Figure 10:**
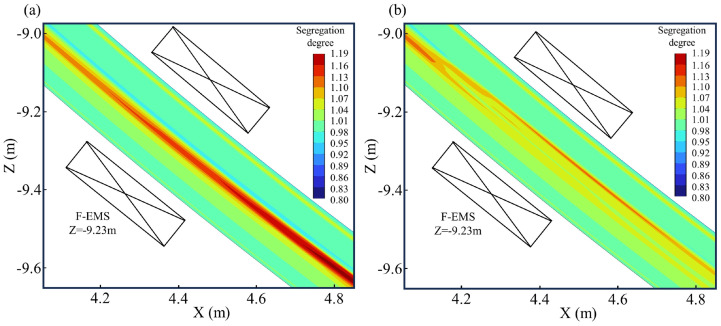
Effect of F-EMS on carbon distribution: (**a**) without F-EMS; (**b**) F-EMS at 250A-6Hz.

The carbon distributions near the F-EMS region are illustrated in Figure 10. Figure 10a shows that in the absence of EMS, carbon tends to accumulate significantly in the liquid phase pool during the final stages of solidification. After complete solidification, carbon in the central region cannot diffuse, resulting in severe central macrosegregation issues on the cross-section, as indicated by the carbon distribution cloud map after complete solidification in Figure 4c. Figure 10b depicts the carbon distribution cloud map on the longitudinal section when there is no M-EMS, and F-EMS operates at 250A-6Hz. It is evident that F-EMS significantly improves the solute distribution in the liquid phase pool during the final stages of solidification. The stirring effect from F-EMS contributes to a more uniform distribution of solute in the liquid phase pool. It is worth noting that F-EMS, as revealed in this study, has a pronounced improvement effect on central macrosegregation issues, which differs from the results obtained by Wang et al. [26]. This disparity can be attributed to the appropriate installation of F-EMS in this study at a position where carbon solutes accumulate significantly. In Wang et al.’s study, the F-EMS action region was too forward (approximately Z = −10.2 m), while, based on their solute distribution results, the location where carbon accumulated significantly during the final stages of solidification was approximately Z = −15 m. This improper installation of F-EMS in Wang et al.’s study might have led to suboptimal stirring effects, allowing solutes to still accumulate significantly during the final stages of solidification, resulting in central macrosegregation.

**Figure 11:**
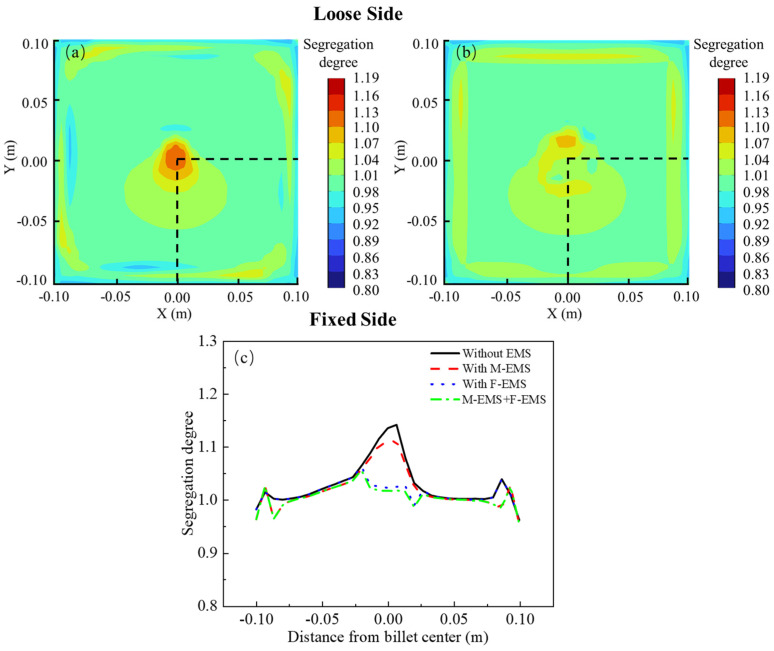
The effect of different EMS modes on carbon distribution: (**a**) M-EMS only; (**b**) F-EMS only; (**c**) comparison of segregation degree.

**Figure 12:**
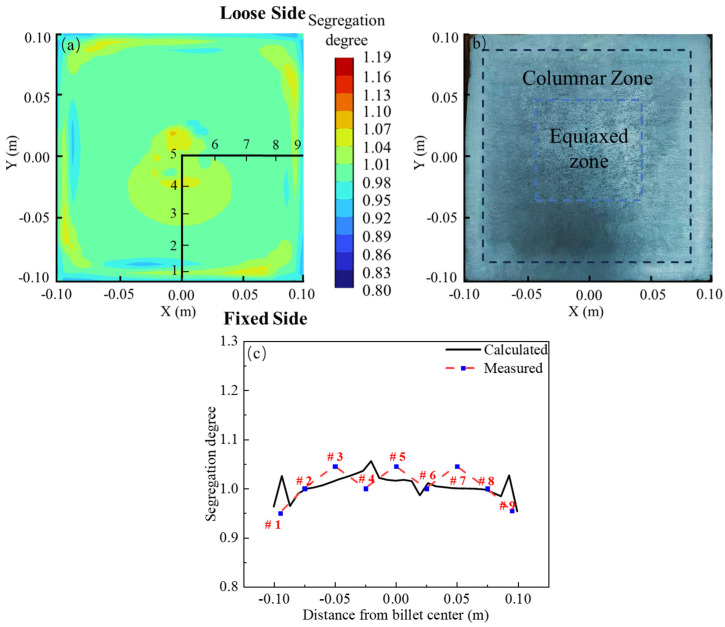
Carbon segregation under M+F-EMS: (**a**) simulation result; (**b**) macrostructure photo of the test billet; (**c**) comparison of simulation and experimental results.

In the cross-section at Z = −11 m, where the billet has completely solidified, the carbon distributions under different EMS modes are illustrated in Figure 11. The parameters for M-EMS are set at 250A-2Hz, while F-EMS operates at 250A-6Hz. Figure 11a depicts the carbon segregation with only M-EMS. A noticeable center segregation issue persists, indicating that the effectiveness of M-EMS in addressing the center segregation problem is limited. Figure 11b shows the carbon distribution with only F-EMS. The introduction of F-EMS significantly reduces the carbon concentration in the central liquid pool, with even a slight negative segregation at the edges of the center. Figure 11c compares the carbon segregation indices under different agitation modes, with sampling locations consistent with those in Figure 11a,b. From the graph, it is evident that without EMS, the center segregation problem is most severe, with the maximum carbon segregation index along the sampling line reaching 1.15. With only M-EMS, besides changes in the carbon distribution within the initial solidification shell formed in the mold, there is a reduction in carbon concentration at the center, yielding a maximum carbon segregation index of approximately 1.11, a decrease of 0.04 compared to the case without EMS. In the case of only F-EMS, aside from a significant reduction in carbon concentration within the central liquid pool, there is no change in the carbon distribution at other locations. At this point, the carbon segregation index at the center is approximately 1.02, indicating a noticeable improvement in the center segregation problem. The impact of M+F-EMS on carbon segregation is presented in Figure 12.

Figure 12 illustrates the carbon segregation distribution in the billet when both M-EMS and F-EMS are operational, with M-EMS and F-EMS parameters set at 250A-2Hz and 250A-6Hz, respectively. In Figure 12a, the simulated carbon segregation after complete solidification shows the impact of M-EMS generating a negative segregation band and F-EMS improving the center segregation. The central liquid pool distribution is more uniform in comparison to Figure 11b. Experimental trials with M+F-EMS were conducted in a steel plant, with M-EMS operating at 250A-2Hz and F-EMS at 250A-6Hz, mirroring the simulation parameters. The macrostructure photograph of the test billet is presented in Figure 12b. The size distribution of the fine crystal zone at the billet edge, the intermediate columnar zone, and the central equiaxed zone are similar to the case without EMS. However, no significant point segregation is observed at the center. Using the same sampling method, the experimental carbon segregation results are compared with the simulation in Figure 12c. The carbon segregation degree at the center in the experiment is 1.05, closely aligning with the simulated value of 1.02. The experimental and simulated results are in good agreement. Combining the findings from Figure 9 and Figure 10, it is evident that M-EMS alters the carbon distribution in the initial solidification shell within the mold, generating a negative segregation band approximately 15 mm from the edge due to the effect of the circulation. M-EMS has a certain improvement effect on the center segregation issue. F-EMS significantly improves center segregation, and with M+F-EMS, the carbon distribution on the cross-section of the billet is more uniform than with only F-EMS.

## 5. Conclusions

(1) Both M-EMS and F-EMS induce tangential flow in molten steel, influencing the solidification and solute distribution processes within the billet. When M-EMS operates at 250A-2Hz, the maximum tangential velocity occurs at the periphery of the liquid pool, causing a rotational flow that deviates the shape of the liquid pool in the mold. For F-EMS operating at 250A-6Hz, the maximum tangential velocity is observed at $f_{l}=0.7$, and F-EMS accelerates heat transfer in the liquid pool, reducing the central liquid fraction from 0.93 to 0.85.
(2) Both M-EMS and F-EMS alter the solute distribution within the billet. M-EMS, by increasing the tangential velocity of the steel, enhances the scouring effect of the molten steel on the solidification front, forming a negative segregation band in the mold. In comparison, F-EMS has a more pronounced effect on alleviating central segregation issues. When F-EMS operates at 250A-6Hz, the central carbon segregation is reduced from 1.15 to 1.02, demonstrating a more significant improvement.
(3) The model was validated through experiments in a steel plant. In the absence of EMS, both experimental and simulated results yielded a central carbon segregation of 1.15 and 1.13, respectively. With M+F-EMS in operation, the central carbon segregation decreased to 1.05 in experiments and 1.02 in simulations. Both experimental and simulated results indicate that M+F-EMS promotes a more uniform solute distribution in the cast billet, with a noticeable improvement in central segregation.
(4) Based on the simulation results, it can be inferred that the effect of M-EMS on improving central segregation is not significant, while F-EMS shows a remarkable improvement. In comparison with previous findings, it can be considered that the installation position of F-EMS might be closely related to the effectiveness of improving central segregation. Subsequent studies should delve deeper into the analysis of this issue.

## Figures and Tables

**Table 1:** Three-dimensional geometric model parameters.

Parameters	Value
Billet cross-sectional dimensions	200 mm × 200 mm
Continuous casting machine arc radius	12 m
Mold effective length	0.8 m
SEN inner diameter	0.026 m
SEN outer diameter	0.09 m
SEN depth	0.11 m
Mold thickness	0.01 m
Vertical distance from M-EMS to meniscus	0.45 m
Vertical distance from F-EMS to meniscus	9.23 m

**Table 2:** Lengths and water flow rate for each segment in the secondary cooling zone.

	Zone 1	Zone 2	Zone 3	Zone 4	Zone 5
Length (m)	0.3	1.0	1.3	1.5	1.5
Water flow rate (L/min)	43.9	38.4	23.3	17.8	13.7

**Table 3:** Chemical composition of 20# steel.

Element	C	Si	Mn	S	P	Cr
Concentration (wt.%)	0.17–0.23	0.17–0.30	0.35–0.65	<0.035	<0.035	<0.25

**Table 4:** Properties of 20# steel.

Parameter	Symbol	Value
Thermal conductivity of liquid phase (W/(m·K))	$k_{T,l}$	38
Thermal conductivity of solid phase (W/(m·K))	$k_{T,s}$	40
Density of the billet (kg/m^3^)	ρ	7100
Viscosity (Pa·s)	μ	0.0035
Initial solute concentration of carbon (wt.%)	$C_{0}$	0.20
Latent heat (kJ/kg)	L	270
Solute expansion coefficient (1/wt.%)	$\beta_{C}$	0.011
Thermal expansion coefficient (1/K)	$\beta_{T}$	1.0 × 10^−4^
Diffusion coefficient in the liquid phase (cm^2^/s)	$D_{l}$	0.0761exp(−134,557.44/RT)
Diffusion coefficient in the solid phase (cm^2^/s)	$D_{s}$	0.0052exp(−11,700/RT)
Equilibrium distribution coefficient for carbon	$k_{C}$	0.34
Specific heat of the liquid phase (J/(kg·K))	$c_{p,l}$	828.33
Specific heat of the solid phase (J/(kg·K))	$c_{p,s}$	722
Emissivity of the strand	$\varepsilon_{s}$	0.8

**Table 5:** Experimental production parameters.

Parameter	Value
Pouring temperature (K)	1808–1818
Casting speed (m/min)	1.3–1.4
Pouring carbon content (wt.%)	0.19–0.21
M-EMS operating parameters (A-Hz)	250-2
F-EMS operating parameters (A-Hz)	250-6

## Data Availability

The raw data supporting the conclusions of this article will be made available by the authors on request.
